# Estrogens in Human Male Gonadotropin Secretion and Testicular Physiology From Infancy to Late Puberty

**DOI:** 10.3389/fendo.2020.00072

**Published:** 2020-02-25

**Authors:** Gabriela Guercio, Nora Saraco, Mariana Costanzo, Roxana Marino, Pablo Ramirez, Esperanza Berensztein, Marco A. Rivarola, Alicia Belgorosky

**Affiliations:** ^1^Endocrinology Department, Hospital de Pediatría “Prof. Dr. Juan P. Garrahan”, Buenos Aires, Argentina; ^2^Research Institute Garrahan-CONICET, Hospital de Pediatría “Prof. Dr. Juan P. Garrahan”, Buenos Aires, Argentina; ^3^Facultad de Medicina, Department of Cellular Biology and Histology, Universidad de Buenos Aires, Buenos Aires, Argentina

**Keywords:** cP450arom, ArKO, ERα, ERβ, ERKO, human male reproduction

## Abstract

Several reports in humans as well as transgenic mouse models have shown that estrogens play an important role in male reproduction and fertility. Estrogen receptor alpha (ERα) and beta (ERβ) are expressed in different male tissues including the brain. The estradiol-binding protein GPER1 also mediates estrogen action in target tissues. In human testes a minimal ERα expression during prepuberty along with a marked pubertal up-regulation in germ cells has been reported. ERβ expression was detected mostly in spermatogonia, primary spermatocytes, and immature spermatids. In Sertoli cells ERβ expression increases with age. The aromatase enzyme (cP450arom), which converts androgens to estrogens, is widely expressed in human tissues (including gonads and hypothalamus), even during fetal life, suggesting that estrogens are also involved in human fetal physiology. Moreover, cP450arom is expressed in the early postnatal testicular Leydig cells and spermatogonia. Even though the aromatase complex is required for estrogen synthesis, its biological relevance is also related to the regulation of the balance between androgens and estrogens in different tissues. Knockout mouse models of aromatase (ArKO) and estrogen receptors (ERKOα, ERKOβ, and ERKOαβ) provide an important tool to study the effects of estrogens on the male reproductive physiology including the gonadal axis. High basal serum FSH levels were reported in adult aromatase-deficient men, suggesting that estrogens are involved in the negative regulatory gonadotropin feedback. However, normal serum gonadotropin levels were observed in an aromatase-deficient boy, suggesting a maturational pattern role of estrogen in the regulation of gonadotropin secretion. Nevertheless, the role of estrogens in primate testis development and function is controversial and poorly understood. This review addresses the role of estrogens in gonadotropin secretion and testicular physiology in male humans especially during childhood and puberty.

## Introduction

The role of the hypothalamic-pituitary-gonadal (HPG) axis and the endocrine system in the control of both spermatogenesis and testosterone production in males is well-known. The reproductive system is coordinated by the HPG axis. Hypothalamic gonadotropin-releasing hormone (GnRH) neurons represent the final common pathway for neuronally derived endogenous as well as exogenous stimuli (1). The anterior pituitary gland secretes gonadotropins, follicle-stimulating hormone (FSH), and luteinizing hormone (LH) in response to the hypothalamic GnRH signal. Gonadotropins stimulate the gonads to produce sex steroids and gametes. GnRH and gonadotropin secretion are modulated by sex steroids acting through their receptors -estrogen (ER) and androgen (AR) receptors- on the hypothalamus and the pituitary gland by way of feedback-regulating mechanisms (2–4). The role of the estradiol-binding protein (GPER) at the hypothalamic and pituitary level remains to be elucidated (5). The aromatase enzyme (cP450arom), which converts androgens to estrogens, is widely expressed in human tissues (including gonads and hypothalamus) even during fetal life, suggesting that estrogens are also involved in human fetal physiology. During pregnancy, androgen precursors are produced by the fetal adrenal glands (6–8).

Knockout (KO) mouse models for aromatase (ArKO) and estrogen receptors (αERKO and βERKO) are useful tools for understanding the role of estrogens in the regulation of the HPG axis. In addition, patients with aromatase deficiency and deleterious ER variants represent a model of nature that may be helpful to explain how estrogens act in the human reproductive system and gonadal physiology from infancy through adulthood.

The role of estrogens in primate testis development and function is still poorly understood. Therefore, in this chapter we discuss the role of estrogens in gonadotropin secretion and testicular physiology.

## Estrogen Synthesis. Cytochrome P450 Aromatase

Estrogens control many physiological processes in mammals, including reproduction, spermatogenesis, ovulation, granulosa-cell proliferation, reproductive tract development, breast development, cardiovascular health, and bone integrity. Biosynthesis of estrogens from androgens is catalyzed by cP450arom, an enzyme located in the endoplasmic reticulum of estrogen-producing cells. This enzyme, a member of the cytochrome P450 superfamily, is expressed in different human tissues, such as the central nervous system, gonads, adipose and bone tissues, among others, and fetal tissues, such as liver, skin, intestine, testis, and ovary, as well as the syncytiotrophoblast layer of the placenta (9).

**Figure 1:**
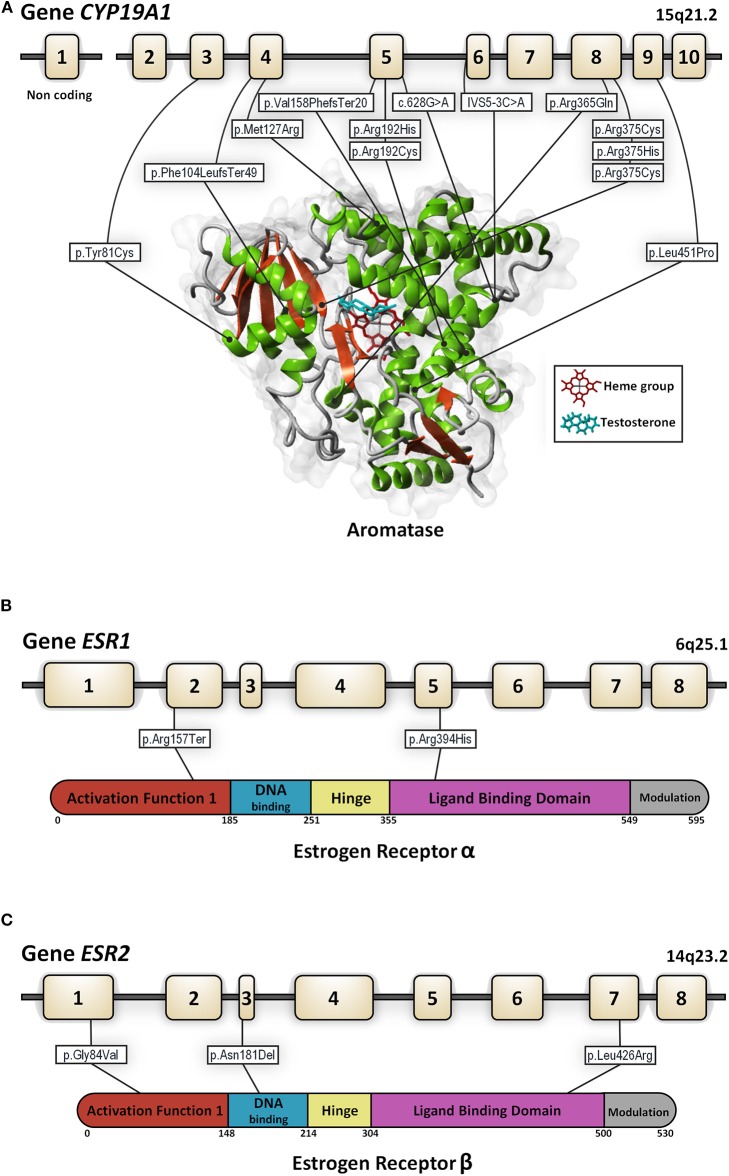
Genomic organization for the *CYP19A1* **(A)**, *ESR1* **(B)**, and *ESR2* **(C)** genes. In each panel structure of the gene and the protein with its functional domains is presented as well as all the mutations described to date in 46, XY subjects (in white rectangles). Numbered boxes represent exons. Lines represent introns. For the aromatase enzyme the *in-silico* 3D model was created using the structure factor file containing the X-ray crystallographic structure of human placental aromatase cytochrome P450 (*CYP19A1*) complexed with testosterone (PDB ID: 5JKW) (doi: 10.2210/pdb5JKW/pdb). The 3D models were created and viewed using YASARA software (©1993–2018 by Elmar Krieger, www.yasara.org) with FoldX Suite plugin.

Human cP450aromis encoded by a single gene, the *CYP19A1*, that maps to chromosome 15q21.1 and contains nine coding exons (E2-E10) spanning ~35 kb (10). There is a tissue-specific regulation of the aromatase gene expression by promoter regions related to several different first exons; however, a unique aromatase protein sequence is expressed in every tissue as these exons are not translated (Figure 1A).

## Estrogen Actions. Human Estrogen Receptors

Estrogen actions are mainly mediated through ERs, alpha (ERα), and beta (ERβ), which belong to the steroid receptor superfamily (11, 12) and are encoded by the *ESR1* and *ESR2* genes, respectively (Figures 1B,C). Both receptors contain six functional domains: the activation function (AF1) domain that contains the N-terminal transactivation domain, a target for phosphorylation, a DNA binding domain, a hinge region, a ligand-binding domain, and a modulation region (13). Both receptors are highly homologous in DNA and ligand-binding domains and divergent in other regions (14). The *ESR1* gene consists of eight exons spanning more than 140 kb. The *ESR2* gene contains eight exons as well. Both the *ESR1* and *ESR2* genes are subject to alternative splicing with the use different start sites and although their exon and functional domain organization is similar, the splice variant isoforms identified are distinct and have been implicated in tissue-specific patterns of gene expression.

These nuclear receptors act as transcriptional regulators through their direct interaction with specific co-regulators (15). The recruitment of different co-regulators in large multifunctional protein complexes is intrinsic to estrogen signaling which is involved in chromatin remodeling, histone modifications, transcription initiation and elongation, splicing, and proteasomal degradation. Therefore, co-regulators might be associated with either activation or repression of transcription. However, there are completely distinct cellular responses by ER subtype-specific and non-specific agonists. In this line, it has been reported that ERβ might exert an inhibitory effect on ERα-mediated signaling or an opposite effect in target tissues (16). Moreover, it has been reported that ERα and ERβ differentially mediated the regulation of miRNA expression (17–19). The AF1 domain in ERα is very active while the activity of the AF1 domain of ERβ is negligible. Interestingly, different responses of both receptors to synthetic antiestrogens have been reported. In this context, a better clinical outcome was proposed in tamoxifen-resistant ER-expressing breast tumors when high expression of ERβis found (20). A study by Lindberg et al. (21) showed that the expression of ERβ in breast cancer tissue reduced AKT activation and upregulated PTEN expression increasing sensitivity to tamoxifen therapy.

ERα is expressed in both the nucleus and the plasma membrane. The non-genomic or membrane action of estrogens exerts rapid membrane-cell signaling effects influencing gene transcription through membrane ERα and/or GPER1 (22–24). Moreover, some studies have shown a role of ERα in the mitochondria (25). The localization of estrogen receptors in the mitochondrial is tissue specific. However, its effects are not restricted to the presence inside the mitochondria. The major role of estrogens via ERs, mainly ERβ, is related to the control of mitochondrial biogenesis and quality control as well as antioxidant defenses (26, 27). In addition, it has been shown that the alteration of ER and GPR30 signaling induces mitochondrial dysfunction affecting testicular steroidogenesis and spermatogenesis (28, 29).

Tissue-specific expression of ERs has been reported. Specifically, ERα is highly expressed in the uterus and pituitary tissues with lesser expression in bone, liver, hypothalamus, mammary gland, vagina, and adult human testes (13, 22, 30) while ERβ expression is high in granulosa cells, prostate (31), and immature and adult human testes (32, 33).

## Knockout Mouse Models for Aromatase and Estrogen Receptors

### The Aromatase-Knockout Mouse

Murine aromatase is expressed in Sertoli cells of immature and in Leydig cells of adult rodents, as well as in spermatocytes and in round and elongated spermatids (34). An aromatase knockout (ArKO) model was presented by Fisher et al. (35). The newborn mice homozygous for the disrupted aromatase gene were described as phenotypically normal. At ~12–14 weeks of age, the internal anatomy of the male ArKO mice showed increased weight of the seminal vesicles (caused by increased volume of secretions) and of combined urinary bladder/prostate but no difference in testicular weight was found. Testosterone (T) levels varied among male mice; however, there was a trend toward overall higher levels compared to wild-type (WT) mice. Androstenedione concentration was also variable among animals, but several of them had values at least twice as high as values reported for the WT males. In the ArKO males, serum LH levels were elevated while serum FSH levels were normal. Additionally, Leydig-cell hyperplasia/hypertrophy was observed in the male ArKO mice, presumably because of high circulating LH levels. The ArKO mice showed normal fertility when young, although with advancing age they developed progressive infertility, as a decreased rate of litter siring became evident. Between 4.5 months and 1 year, these mice exhibited disruptions of spermatogenesis with increased frequency of germ-cell apoptosis and defects in spermiogenesis with the epididymis showing reduced or complete absence of sperm (36). These findings suggest that estrogen plays a role in the neuroendocrine regulation of LH secretion and in spermatid differentiation and spermatogenesis.

Recent studies in ArKO as well as αERKO mice have suggested that estrogens play a role in the normal development of the penis. The male ArKOmice, that completely lack estrogen production, presented with a mild hypospadias phenotype similar to that reported in the male αERKO mice (37).

### Estrogen Receptor Knockout Mouse

Both estrogen receptor ERα and ERβ are expressed in the efferent ducts and epididymis, while ERα is found in Leydig cells and ERβ in spermatocytes in the developing mouse testis (30, 38).

The first αERKO mouse was developed by disruption of the mouse ER gene in 1993 (39). In these αERKO mice, decreased fertility was observed. However, subsequently the male offspring were found to be heterozygous αERKO mice. A new study in confirmed homozygous male αERKO mice revealed that these mice were completely infertile (40). They showed complete disruption of the seminiferous tubules and a significant reduction of testis weight. In adulthood, the lumen of all the seminiferous tubules were considerably enlarged and sperm quantity and quality was affected. The disrupted phenotype seemed to be more profound in older males. These findings suggest a progressive degenerative process. Fluid collection within the lumen of the seminiferous tubules that increased intratesticular pressure affecting the testicular blood flow was described. Serum testosterone levels were moderately but significantly higher, and serum LH and serum FSH were slightly higher than in the WT mice. In the male αERKO mice, behavioral aspects of reproduction were also affected reflected in decreased intromissions and weak ejaculation as well as significantly reduced aggressive behavior (41). Additionally, sperm from αERKO mice was ineffective at *in vitro* fertilization, suggesting that not only production but also function of spermatozoa was affected. Nevertheless, male WT mouse depleted of germ cells and transplanted with germ cells from male αERKO mouse produced fertile sperm, demonstrating that spermatogenic stem cells were not affected (42).

Another αERKO male mouse was generated with the deletion of exon 3 (a region encoding the DNA-binding domain), ExαERKO (43), presenting with the same phenotype as that seen in male αERKO mice (44). However, neither serum LH nor serum FSH levels were different from those in WT mice. Therefore, it could be proposed that estrogen receptor activation via a non-genomic pathway might be involved in the neuroendocrine regulation of gonadotropin synthesis and/or secretion.

The generation of the first βERKO mice (45) produced a completely different male phenotype compared to that of αERKO mice. Male βERKO mice were fully fertile, with some age-related abnormalities in the prostate and bladder. Later on, a true ERβ-null mouse was generated, in which exon 3 was deleted through a Cre/LoxP-system and no transcript of ERβ could be detected (46). In this ERβ-null mouse, testis as well as epididymis histology were normal, and the motility of their spermatozoa resemble normal sperm; however, mating defects were reported. It was concluded that both female and male ERβ-null mutants are sterile; however, the origin of the sterility in males is unknown.

Male mice lacking ERα and ERβ, αβERKO, showed a phenotype similar to that of male αERKO mice (31). Male αβERKO mice are infertile but have a generally normal reproductive tract. The number and motility of epididymal sperm was reduced. The most remarkable characteristic of αβERKO mice is seen in female adult mice, in which the ovaries show follicular differentiation to structures resembling seminiferous tubules of the testis, including Sertoli-like cells and expression of Müllerian inhibiting substance (MIS), sulfated glycoprotein-2, and Sox9. Elevated Sox9 mRNA levels were also detected in the testes of adult αβERKO and αERKO males (31, 47). It is well-known that very early in gestation, the expression of the transcription factor SOX9 in the bipotential gonad of the 46,XY fetus is required to induce testicular cell-fate differentiation (48). This finding raises the question of whether lack of expression of both ERα and ERβ in the bipotential gonad in 46,XY fetus is mandatory to allow for the expression of SOX9.

### Membrane and Nuclear αERKO Mouse Models

ERs are predominantly nuclear and cytoplasmic; however, around 5–10% of ERα is found in the cell membranes. In order to define the role of membrane ERα in mouse physiology two mouse models were developed. One with membrane-only ERαexpression (MOER) and another with nuclear-only ERα expression (NOER) (49, 50). Both transgenic mice presented with male infertility and sperm abnormalities (51). However, no description about the reproductive tract of the male MOER mouse has been published.

Male NOER mice present with many abnormalities that are similar to those of male αERKO mice, such as decreased sperm production with impaired sperm motility and viability. Testis weight of NOER mice became greater than that of WT mice at 4 months of age when many of the seminiferous tubules were degenerated. Nevertheless, at 8 months of age, when profound histological changes in testis, rete testis, and efferent ducts were found in NOER mice, testis weight was not different from that of WT mice. At that time, NOER males showed great structural abnormalities in cauda epididymal sperm, similar to male αERKO mice. Serum T levels in NOER mice were significantly higher than in WT mice, while serum LH, FSH, and E2 levels were not different. Similar to male αERKO mice, NOER males become infertile with advancing age; however, juvenile NOER males are transiently fertile before tubule abnormalities become evident. The phenotype of male NOER mice may be considered less severe as it develops more slowly than that of male αERKO mice (52).

A comparison of the significant clinical phenotypes and hormonal studies in all KO-mice models is shown in Table 1.

**Table 1:** Phenotypes of knockout male mouse models.

	**ArKO**	**αERKO**	**βERKO**	**αβERKO**	**NOER**	**MOER**
Fertility	Initially fertile although with advancing age they developed progressive infertility.	Infertile	Fertile/Infertile^*^	Infertile	Initially subfertile, becoming infertile with advancing age	Infertile
**Gonadotropins**						
Serum LH	Elevated	Not significantly higher	NA	NA	Normal	NA
Serum FSH	Normal	Not significantly higher	NA	NA	Normal	NA
**Serum Steroids**						
Androgens	Tendency toward elevated levels	Elevated	NA	NA	Elevated	NA
Estrogens		Normal	NA	NA	Normal	NA
Testicular function	At ~12–14 weeks of age the internal anatomy of the male ArKO mice showed increased weight because of the seminal vesicles (caused by increased volume of secretions) and combined urinary bladder/prostate but no difference in the testes weight. Leydig cell hyperplasia/hypertrophy	Testis weight significantly reduced. Disruption of seminiferous tubules.	Normal with some age-related abnormalities in the prostate and bladder	Showed similar phenotype as male αERKO mice.	At 4 months of age seminiferous tubular degeneration. At 8 months of age: significant histological alterations in testis, rete testis, and efferent ductus.	NA
Sperm	Disruptions in spermatogenesis with increased frequency of germ cell apoptosis and defects in spermiogenesis with the epididymis showing reduced or complete absence of sperm	In adulthood quantity and quality of sperm affected. Spermatogenic stem cells not affected	Normal	Similar to male αERKO mice	Decreased sperm production with impaired sperm motility and viability	NA

* *AnERβ mouse mutant generated by Cre/LoxP deletion of exon 3; reported impaired mating, suggesting male infertility (46)*.

*NA, Not Available*.

## Aromatase and Estrogen Receptor Expression in Human Testis From Infancy to Adulthood

### Aromatase

In the human testis, aromatase is mainly expressed in interstitial, in fetal and adult Leydig, and germ cells. Nevertheless, the strongest aromatase immunostaining has been reported in neonatal and infant testes (Table 2) (30, 53, 54). Therefore, in human testes, in order to maintain an intratesticular balance between androgens and estrogens, it could be proposed that a short loop regulation of aromatase expression might occur mainly when postnatal testicular activation takes place. In addition, peritubular, and Sertoli cells showed weak and persistent aromatase expression without changes throughout the testicular maturation (33). In adult testes, aromatase is expressed in immature germ cells and in ejaculated spermatozoa, suggesting that aromatase might be involved in sperm motility. In this line, the fact that in 46, XY aromatase-deficient patients an alteration in the spermatogenesis related to sperm count and motility was reported, it could be speculated that estrogen action is required for immature germ-cell maturation and fertilizing capacity (33, 55, 56).

**Table 2:** Expression of P450arom, ERα, and ERβ during postnatal maturation in human testis (33).

		**Interstitial cells**	**Leydig cells**	**Peritubular cells**	**Sertoli cells**	**Germ cells**
P450arom	Neonate	**XX** (37)	**XXX** (71)	**X** (13)	**-** (4)	**XX** (31)
	Infancy	**XX** (41)	**XX** (25)	**XX** (18)	**X** (8)	**XX** (27)
	Childhood	**X** (19)	**Nd**	**X** (12)	**-** (3)	**X** (17)
	Puberty	**-** (0)	**-** (0)	**-** (0)	**X** (9)	**XXXX** (96)
ERα	Neonate	**-** (0)	**-** (2)	**-** (0)	**-** (0)	**-** (0)
	Infancy	**-** (0)	**-** (2)	**-** (0)	**-** (0)	**-** (0)
	Childhood	**-** (1)	**Nd**	**-** (2)	**-** (0)	**-** (0)
	Puberty	**X** (6)	**XXXX** (97)	**-** (1)	**-** (0)	**-** (0)
ERβ	Neonate	**XX** (26)	**-** (4)	**X** (15)	**X** (24)	**XX** (45)
	Infancy	**X** (12)	**X** (12)	**X** (13)	**XX** (26)	**XX** (42)
	Childhood	**X** (12)	**X** (12)	**X** (9)	**XX** (44)	**XXX** (52)
	Puberty	**X** (21)	**X** (16)	**-** (0)	**XXX** (74)	**XXXX** (94)

*Immunostaining studies expressed as range of percentage of positive cells with line or X and in brackets representing percentage of positive cells*.

*(**-**): 0–5%; (**X**): 5–25%; (**XX**): 25–50%; (**XXX**): 50–75%; (**XXXX**): 75–100%*.

*Nd, Not determined*.

### ERα and ERβ in Primate Testes

We found that ERβ (Table 2) is the predominant form of ER expressed in human testes of neonates and infants (33). Different variants of ERβ have been reported, e.g., ERβcx, also known as ERβ2, a truncated variant with the loss of 61 aminoacids at the C-terminus. Expression of ERβ1 and ERβ2 is described in the human testis: ERβ1 was expressed in somatic cells, in pachytene spermatocytes and in round spermatids, while ERβ2 was expressed in fetal gonocytes (22, 57). In gonocytes and spermatogonia, ERβ was strongly immunoexpressed, while no expression of ERα was detected in germ cells. The spermatogonia might be a target of estrogens in prepuberty and adulthood. Positive expression of ERα in postmeiotic germ cells was found in puberty (33). Although the expression of ERβ in human testes increases during puberty, it is already high during childhood, when serum gonadotropins are very low or absent (33). Interestingly, even though there is no information about the role of ERβ target genes in human neonatal testis, it has been reported that in a mouse model with homozygous or heterozygous inactivation of ERβ, very soon after birth the number of gonocytes increased secondary to decreasing cell apoptosis. Nevertheless, the number of Sertoli and Leydig cells was not modified. It has been proposed that gonocytes are very sensitive to estrogens via the ERβ pathway during the neonatal period. Exposure to environmental endocrine disruptors (xenoestrogens) during fetal and neonatal testicular development might therefore lead to fertility disorders in adulthood (58). In adult human testicular biopsies, Cavaco et al. (32) showed that ERα and ERβ are expressed in somatic and germinal testicular cells. Immunoexpression of ERα was present in Leydig cells and Sertoli cells, as well as in immature and post-meiotic germ cells, while ERβ was expressed in the same cell types but not in spermatogonia and Sertoli cells, suggesting that both ER isoforms are involved in testicular function (32).

Lambard et al. (55) reported ERβ proteins (full-length and variant) in human male germ cells, but the possible mechanism of action of estrogens in these cells, in terms of both genomic and “non-genomic” pathways, remains to be elucidated (55).

As shown in our study (Table 2), in the immature human testes ERβ expression was higher in neonatal than in infant and juvenile testes. Interestingly, in newborns a high testicular growth rate mediated by decreased apoptosis was observed (59). In human and monkey testes, we have found minimal ERα expression in germ cells during prepuberty, but during puberty a marked upregulation of ERα was found in germ cells of both monkeys and humans (60).

A growing number ERβ splice variants have been reported. In haploid germ cells, high levels of novel ERβ deletion variants were found. The cell-specific distribution raises the question of whether the differential splicing of ERβ is regulated in a cell-specific manner and whether ERβ play a specific role in spermatogenesis (61).

Interstitial cells expressed ERβ, particularly in the neonatal and infant periods with a weaker expression during childhood, while ERα expression was <5% at all ages. Interstitial cells might be targets of estrogens during prepuberty and adulthood (33). During testicular development, three growth phases of Leydig cells have been reported in humans. In the first phase the cells are named “fetal Leydig cells” and they synthesize testosterone under placental βhCG stimulation, which is necessary for fetal masculinization, and insulin-like 3 factor for testicular descent. During infancy, testicular “infant Leydig cells” are involved in the production of testosterone under LH stimulation. This phase is known as the postnatal pituitary-testicular activation period (minipuberty). The last phase of “adult Leydig cells” coincides with pubertal development under LH regulation (33).

In neonatal and infant Leydig cells ERß staining was weak and during childhood it was even weaker. ERα staining was very weak in all cell compartments at any age in human prepubertal testicular tissue, but it was strong in the efferent ductules of the same samples (41) (Table 2). Similarly, no immuno expression of ERα was detected in juvenile primate testes, while strong expression was seen in the efferent ductules (22, 62).

Finally, although robust evidence is available on the role of estrogens in testis physiology during the life span, further studies, mainly in the human testes, are necessary.

## Role of Estrogens in Gonadotropin and Testicular Physiology in Humans

Evidence suggests that in adult males, estrogens exert a negative feedback both at the hypothalamic and the pituitary level. The exogenous administration of estradiol to normal men and patients with isolated GnRH-deficiency was associated with significant decreases in serum gonadotropins as well as LH pulse amplitude (63) suggesting a direct pituitary effect of estrogens. Additionally, lack of gonadotropin suppression was reported when a non-aromatizable androgen was used (64–66).

The relevance of estrogen action in the hypothalamus is supported by the effect on serum gonadotropin levels with the use of the selective aromatase inhibitor anastrozole in normal men and men with idiopathic hypogonadotropic hypogonadism (IHH) after long-term pulsatile GnRH therapy (65, 66). Anastrozole led to an increase of mean serum LH and FSH levels in IHH and normal men. Moreover, greater increases in serum gonadotropin levels were observed in normal subjects, suggesting that an intact hypothalamus is needed for the full effect of the aromatase inhibitor.

Additionally to estradiol regulation, the product of Sertoli cells, inhibin B, participates in the negative feedback of FSH as shown mainly by animal studies (67). In normal men, however, the dynamics of the relationship between serum inhibin B and FSH levels varies according to pubertal development. At midpuberty, maturational changes in the negative feedback regulation of the HPG axis occur and persist into adulthood. In this process, estradiol is likely to play an important role (68). Therefore, it has been proposed that estrogen administration to boys during early and midpuberty would decrease serum LH concentration and LH pulse frequency but would have no effect on LH pulse amplitude (69). In the same line, it was reported that the use of letrozole in normal boys increased serum LH levels, LH pulse amplitude, and GnRH-induced LH during early and mid-puberty (70).

### Human Models of Estrogen Deficiency and Resistance

The identification and characterization of human patients with mutations in *ESR1* or aromatase allowed for a better understanding of the role of estrogen in the human HPG axis in men (71). Estrogen resistance due to an *ESR1* (estrogen receptor α[ERα]) gene mutation is a rare condition, first described by Smith et al. (72). To date, only five patients with *ESR1* mutations have been reported (46, XY *n* = 2 and 46, XX, *n* = 3 patients) (Figure 1B, Table 3).

**Table 3:** Molecular defects in the *ESR1* gene, *in vitro* activity of mutants, and clinical phenotype in published 46, XY subjects with estrogen resistance due to *ESR1*-mutations.

**Gene Mutations**	**Description**	**Functional analysis**	**Phenotype**	**References**
c.[469C>T];[469C>T]	p.Arg157Ter	Not determined (ND); however, the protein would be severely truncated lacking DNA binding and substrate binding domains.	At Birth: Normal male genitalia, bilateral descended testes. At 28 years: Tall stature, genu valgum. High serum estrogens and gonadotropins. Normal sperm density. Severely undermineralized skeleton.	(72)
c.[1181G>A];[1181G>A]	p.Arg394His	Strongly reduced transcriptional activity and inability to securely anchor the activating hormone, estradiol, compared with wild-type ERα.	At 18 years: Marked delayed bone maturation. Tanner stage I gonadal development with a cryptorchid right testis. Hypoplastic left testis. High serum basal 17β estradiol and gonadotropin levels, and low testosterone levels.	(73)

Two male patients from two consanguineous families with *ESR1* mutations were reported (72, 73). The first patient described by Smith et al. presented at 28 years of age with normal male genitalia and bilateral 20–25 ml descended testes. Serum estrogens (estradiol and estrone) and gonadotropin concentration were high without changes after 6 months of estrogen therapy. Semen analysis revealed normal sperm density. The affected patient described by Bernard et al. (73) showed Tanner stage I pubertal development at 18 years of age with a cryptorchid right testis and a hypoplastic left testis (volume <1 mL). He also had high serum estrogen and gonadotropin levels, but AMH, inhibin B, and testosterone levels were low. In both cases, the variability in the clinical phenotype, hormonal profile, and testicular function was surprising, and it could not be explained by the genotype. Nevertheless, the contribution of the untreated cryptorchidism to the biochemical profile cannot be ruled out. In addition, in both cases bone age was delayed, confirming the role of estrogen in skeletal maturation.

Recently, biallelic and monoallelic *ESR2* variants associated with a 46, XY disorder of sex development and in a 46, XX woman with early-onset complete ovarian failure were reported (74, 75) (Table 4). The 46, XY individuals had variable degree of gonadal dysgenesis. Interestingly, negative ERβ immunostaining was reported in a human 46, XY embryo at 8 weeks of gestational age around the time of sex determination (75). In this line, Kuiper et al. (12) proposed that the relationship between the two receptors is required to determine the different estrogen effects in target tissues. In addition, Baetens et al. (74) has proposed a role for ERβ in early gonadal development or in its differentiation. Nevertheless, as in male αβERKO mice a normal reproductive tract, but in female αβERKO mice sex reversal was observed (31), it could be speculated that in the undifferentiated gonad lack of ER expression is required to induce testicular cell fate. The two patients with more severe forms or complete gonadal dysgenesis presented with high serum FSH levels; however, a role for*ESR2* in the regulation of the HPG axis and gonadotropin secretion cannot be ruled out.

**Table 4:** Molecular defects in the *ESR2* gene, *in vitro* activity of mutants, and clinical phenotype in published46,XY subjects with estrogen resistance due to *ESR2* mutations.

**Gene mutations**	**Description**	**Functional analysis**	**Phenotype**	**References**
c.[541_543del];[541_543del]	p.Asn181del (ACMG: likely pathogenic)	Located in the highly conserved DNA-binding domain. Deleterious effect by structural analysis. Transcriptional activation significantly increased.	At birth: syndromic 46,XY DSD. At 15 years: delayed bone age. Female external genitalia. Absence of uterus, fallopian tubes, gonads, and vagina. High basal gonadotropin levels, undetectable testosterone, AMH and Inhibin B levels	(74)
c.[251G>T];[=]	p.Gly84Val (ACMG: Likely pathogenic)	Located in the N-terminal domain. Structural analysis: ND. No significant alterations of ligand-dependent transcriptional activation.	At birth: non-syndromic 46,XY DSD. At 12.5 years: delayed bone age. Atypical genitalia: clitoromegaly, urogenital sinus. Absence of uterus. High basal gonadotropin levels	(74)
c.[1277T>G];[=]	p.Leu426Arg (ACMG: uncertain significance)	Located in the ligand-binding domain. Deleterious effect by structural analysis. No significant alterations of ligand-dependent transcriptional activation.	At birth: non-syndromic 46,XY DSD. At 24 years: Female external genitalia. Prepubertal uterus. No visible gonads. High basal gonadotropin levels and low testosterone levels.	(74)

Aromatase deficiency is an autosomal recessive disorder first described by Shozu et al. (76) (Table 5). Since then, 43 patients, 31 46, XX and 12 46, XY, have been reported (6).

**Table 5:** Molecular defects in the *CYP19A1* gene, *in vitro* aromatase activity of mutants, and clinical phenotype in published male aromatase-deficient subjects.

**gene mutations**	**Description**	**Aromatase activity**	**Phenotype**	**References**
c.[1123C>T];[1123C>T]	p.Arg375Cys in a highly conserved region.	0.2 % of WT activity. Protein modeling studies suggest that the affected region may be important in anchoring the region of the protein proximal to the substrate access channel to the membrane.	Continuous linear growth, delayed bone maturation, tall stature, eunuchoid body proportions Cisgender, heterosexual, modest libido. Macroorchidism Increased basal gonadotropins and testosterone. Hypertension, Obesity Dyslipidemia, Insulin Resistance Osteopenia	(77)
c.[1094G>A];[1094G>A]	p.Arg365Gln	0.4% of WT activity.	Continuous linear growth, delayed bone maturation, tall stature, eunuchoid body proportion Moderate bone pain. Genu valgum. Cisgender identity and sexual orientation. Normal libido. Infertility Microorchidism Normal basal testosterone, slightly elevated FSH and LH in the upper normal range. Overweight. Dyslipidemia Normal OGTT	(78)
c.[469delC];[469delC]	p.Val158PhefsTer20 C base deletion in exon 5 causing a frame shift and a premature stop codon after 21 codons.	Not determined (ND). However, the resultant peptide does not contain the substrate-binding pocket (I helix), the electron-accepting site and the heme-binding site. An inactive protein would be expected.	At birth: Normal genitalia, and serum AMH levels. Normal serum basal and GnRH stimulated FSH levels at 2 months of age. At 16 years: Tall stature, delayed bone maturation. Normal virilization and testicular volume. High serum basal testosterone, and normal serum basal gonadotropin levels. Osteoporosis.	(79, 80)
c.[628-3C>A];[628-3C>A]	IVS5-3C>A C to A transition at bp−3 at the splicing acceptor site in exon 6. Exon 6 is completely excised leading to a frame shift and premature stop codon 8 nucleotides downstream the end of exon 5.	ND. The resulting peptide most likely will not be processed, but even if it were, it would not result in a functional aromatase because it lacks the substrate-binding pocket, the electron-accepting site and the heme-binding site (81)	Continuous linear growth, tall stature, eunuchoid body proportion. Genu valgum, kyphoscoliosis, pectus carinatus. Cisgender, heterosexual, and normal libido. Normal genitalia and testicular volume Increased serum basal testosterone and FSH, normal serum LH levels. Azoospermia Obesity. Insulin resistance. Osteoporosis.	(81)
c.[628G>A];[628G>A]	G to A transition in the last nucleotide in exon 5. The mutated DNA will generate an mRNA that includes the intron 5 sequence which contains an in-frame stop codon 30 bp downstream the splice junction.	ND. A truncated and inactive protein lacking the heme-binding domain would be expected.	Continuous linear growth, delayed bone maturation, tall stature, eunuchoid body proportions. Diffuse bone pain, genu valgum, Cisgender, heterosexual, referred normal libido Bilateral cryptorchidism (surgery unsuccesful at 6 years). Smal inguinal testes, total germ depletion (biopsy). Normal serum basal LH and testosterone, and increased FSH levels. Overweight, Dyslipidemia. Type 2 diabetes Osteoporosis.	(82)
c.[380T>G];[1124G>A]	p.Met127Arg; p.Arg375His	*In vitro* analysis demonstrated a reduction of aromatase activity when the two mutations were expressed separately or coupled.p.Arg375His showed aromatase activity of 7%. Aromatase activity decreased to 0% when the two mutations were coupled.	Continuous linear growth, delayed bone maturation, tall stature. Diffuse bone pain, genu valgum. Cisgender, heterosexual, normal libido. Normal testicular volume. Normal serum LH and testosterone, increased FSH levels. Focal hypospermatogenesis on testicular biopsy. Obesity, acanthosis nigricans, hepatomegaly. Moderate dyslipidemia, Insulin resistance. Osteoporosis.	(83)
c.[312_334del];[1263+1G>A]	p.Phe312LeufsTer49: 23 bp deletion in exon 4 that would be expected to cause a frame shift with a premature stop codon at nucleotide 361 in exon 4. IVS9+1G>T: point mutation in the first nucleotide of intron 9 that would lead to an aberrant splicing of the mRNA.	A truncated and inactive protein lacking the heme-binding domain would be expected from the c.312_334del allele.	Continuous linear growth, delayed bone maturation, tall stature, eunuchoid body proportions, genu valgum. Cisgender, normal sexual orientation Normal testicular volume. Right cryptorchidism, surgery at 3 years. Mild asteno-teratozoospermia. Increased serum basal FSH with normal LH and testosterone levels. Overweight, Insulin resistance. Mild dyslipidemia Osteoporosis.	(84)
c.[1124G>A];[1124G>A]	p.Arg375His Previously reported (83)		Continuous linear growth, delayed bone maturation, tall stature, eunuchoid body proportions Bone pain, fractures. Macroorchidism Normal serum gonadotropin with increased testosterone levels. Normal sperm count. Overweight. Dyslipidemia. Normal OGTT. Hepatosteatosis. Osteoporosis.	(85)
c.[575 G>A];[575 G>A]	p.Arg192His Amino acid conserved across species and involved in substrate access and catalysis	p.Arg192His mutant was found to have markedly reduced aromatase activity. Both the km and the Vmax were adversely affected. The catalytic efficiency of metabolizing androstenedione was reduced to 19%. Modeling of the structure of the novel p.Arg192His variant of the CYP19A1 protein revealed a crucial role of the arginine 192 residue in substrate binding as well as catalysis.	Mild hypospadias (glans), normal-length phallus, bilateral inguinal testes. Normal serum FSH, LH, testosterone, AMH, and inhibin B levels.	(86)
c.[384A>G];[1494T>C]	p.Tyr81Cys; p.Leu451Pro. Both the Tyr81 and Leu451 residues were highly conserved in vertebrate aromatase orthologs and were also conserved in human aromatase paralogs.	Three-dimensional modeling predicted that the p.Tyr81Cys and p.Leu451Pro mutations would probably result in loss of aromatase function. In-cell aromatase activity assay: p.Tyr81Cys mutant was found to have 14.3% wild-type activity, whereas the p.Leu451Pro mutant was found to have 3.1% wild-type activity. p.Tyr81Cys mutant showed lower Vmax, higher Km, and lower catalytic efficiency. p.Leu451Pro mutant had lower Vmax, Km and catalytic efficiency.	Continuous linear growth, delayed bone maturation, tall stature, eunuchoid body proportions, genu valgum, Normal libido, Normal testicular volume Normal spermogram. Increased serum basal FSH, with normal LH and testosterone levels. Overweight, acanthosis nigricans, Dyslipidemia and severe steatohepatitis. Impaired glucose tolerance and hyperinsulinemia. Osteopenia.	(87)
c.[628 G>A];[628G>A]	Previously reported (88)		Continuous linear growth, delayed bone maturation, unfused growth plate, arachnodactily. Normal libido. Normal testicular volume Normal serum FSH and LH levels with upper normal basal testosterone. Low BMI. Normal OGTT. Osteoporosis	(89)
c.[574C>T];[574C>T]	p.Arg192Cys. Residue highly conserved.	ND SIFT tool predicted this variant to affect protein function with a highly deleterious tolerance index score of 0.00. The mutation was predicted to be probably damaging with a score of 1.000 (sensitivity 0.00; specificity 1.00) using the structure based approach PolyPhen-2.	Accelerated puberty and apparently normal pituitary gonadal function. Pubertal bone mineral accrual was incomplete leading to osteopenia.	(90)

*ND, Not determined; BMI, body mass index; OGTT, oral glucose tolerance test*.

Different deleterious mutations in all exons and intron boundaries of the *CYP19A1* gene were reported in individuals with aromatase deficiency. The mutations described to date in 46, XY patients are shown in Figure 1A.

In aromatase-deficient patients of both sexes, lack of suppression of serum gonadotropin levels into the normal range, along with high serum androgen levels support a major role for estrogens in the mechanism of gonadotropic regulation (6). In most affected men, basal serum LH levels are normal or slightly increased (77, 85); however, dynamic studies showed that LH pulsatility and pulse amplitude are increased (65, 91). Unlike serum LH, serum FSH levels are consistently increased (77, 78, 81–85, 87). Normal serum FSH levels were only reported in two affected males. A direct effect of high serum testosterone levels or residual aromatase activity might explain gonadotropin regulation in these cases (79, 89, 90). In two previously reported adults with aromatase deficiency and severely impaired spermatogenesis, Rochira et al. (91) described the presence of normal-to-low and low serum inhibin B levels along with increased serum FSH concentrations that did not normalize after estrogen replacement. The authors suggested that increments of serum FSH levels were not only related to the lack of estrogen negative feedback, but also to the spermatogenic damage in aromatase-deficient adult men. Nonetheless, in some other affected adult cases, serum FSH levels normalized after estrogen therapy even in the presence of low serum inhibin B levels and asthenozoospermia (84). The paucity of cases reported and the lack of systematic assessment of the HPG axis makes it difficult to validate conclusions regarding the relative contribution of estrogens and inhibin B to the gonadotropic negative feedback. In this line, it has been proposed that serum inhibin B, FSH, and the inhibin B/FSH ratio are useful biomarkers of impaired spermatogenesis (91).

The maturational changes in the dynamics of the regulation of the HGP axis during male pubertal development (see above), might explain the different findings regarding sex steroid-gonadotropin feedback found in adult and prepubertal aromatase-deficient patients. While serum estradiol levels were very low in an affected boy at 2 months of age, normal, and stimulated serum FSH levels were found at later ages. Moreover, serum androgen levels were high postnatally but decreased to the normal range during the first month of life, while serum levels of inhibin B were within the normal range for sex and age (80). The second aromatase-deficient boy identified was born with distal hypospadias and bilateral inguinal gonads (86). Serum FSH, LH, testosterone, AMH, and inhibin B levels were assessed at the age of 4 and 6 years and were within the normal reference range for age and sex. Since this is the only aromatase-deficient male reported with atypical genitalia, and a causative role of aromatase deficiency in the development of male external genitalia was not explored, a relationship between aromatase deficiency and genital phenotype has not been shown. A third boy was recently reported (90). At 8 years of age, the affected patient presented with normal basal and stimulated serum LH and FSH levels along with normal serum inhibin B and AMH concentrations. Taken together, these biochemical findings might suggest that, in contrast to adult men, estrogens do not seem to play a major role in the regulation of the gonadotropin negative feedback mechanism during infancy and prepubertal years in normal boys. Interestingly, the administration of an aromatase inhibitor in a prepubertal boy with idiopathic short stature was not associated with changes in serum gonadotropins (92).

On the other hand, an increase in serum gonadotropin levels along with a very high serum testosterone concentration were found when aromatase inhibitors were used during pubertal years. These findings raise concern regarding pubertal progression (92). In agreement with these observations, during male pubertal development, accelerated progression of secondary sexual characteristics was observed in an aromatase-deficient patient (90). This rapid progression of genital virilization was associated with a significant increase in basal serum testosterone levels and normal basal gonadotropin levels. LH pulsatility and pulse amplitude were not assessed and an increase in one or both parameters could not be ruled out as the underlying mechanism, considering that both effects have been previously demonstrated under estrogen suppression (65).

## Conclusions

This review summarizes estrogen physiology, especially related to its role in the male reproductive system in different periods of maturation, from infancy to young adulthood.

The role of the HPG axis and the endocrine system in the control of both spermatogenesis and testosterone production in males is well-known. GnRH and gonadotropin secretion are modulated by sex steroids acting both on the hypothalamus and the pituitary gland through feedback-regulating mechanisms. Biosynthesis of estrogens from androgens is catalyzed bycP450arom, an enzyme located in the endoplasmic reticulum of estrogen-producing cells. This enzyme, a member of the cytochrome P450 superfamily, is expressed in a number of tissues, including the testes, placenta, and central nervous system.

Estrogen actions are mainly mediated through ERα and ERβ, which belong to the steroid receptor superfamily and are encoded by the *ESR1* and *ESR2* genes. These nuclear receptors act as transcriptional regulators through their direct interaction with specific co-regulators. Estrogens also exert rapid membrane-initiated effects that are known to impact on cell signaling and influence nuclear gene transcription. In addition, the estradiol-binding protein GPR30 also mediates multiple functions in various tissues. Moreover, a role in the mitochondria has been proposed.

ArKO, αERKO, and βERKO mouse models are useful tools for the understanding of the role of estrogens in the regulation of the HPG axis. In addition, patients with affected estrogen synthesis or action represent a model of nature to better understand the role of estrogens in the human reproductive system and gonadal physiology from infancy through adulthood. Male ArKO mouse model studies suggest that estrogen plays a role in the neuroendocrine regulation of LH secretion and in spermatid differentiation and spermatogenesis. Similar to the studies in male ArKO mice, studies in aromatase-deficient adult males but not in affected prepubertal boy simply that estrogen mainly plays a role in the neuroendocrine regulation of LH secretion. Nevertheless, inhibin B might be involved in the FSH negative feedback during childhood, puberty, and adulthood. In addition, a large variability in the number and quality of sperm was reported in the few affected cases studied. The αERKO mouse model showed slight increases in serum gonadotropin and testosterone levels, degeneration of the seminiferous tubules affecting spermatozoa production, including fertilizing capacity. However, in the βERKO null mice model testis and epididymis histology was normal but fertility was affected. The cause of the infertility is largely unknown. Interestingly, and in contrast to mice, ERβ but not ERα is expressed in the immature human testes. In adult human testes both receptors are expressed in somatic and germ cells.

In humans, only two 46, XY patients carrying an ERα gene mutation have been reported. Both patients presented with normal external genitalia but cryptorchidism was observed. Serum gonadotropin, LH, and FSH levels were high confirming the concept that estrogens are required for the regulation of normal negative feedback. Sperm count was normal in one and in the other delayed puberty was observed.

An ERβ gene mutation was reported in three 46, XY patients. Severe sex reversal was observed in all three. The cause of the alteration of sex determination in these 46, XY cases is poorly understood. Interestingly, at 8 weeks of gestational age, around the time of sex determination, negative ER-β immunostaining was reported in a human 46, XY embryo. Nevertheless, as in male αβERKO mice a normal reproductive tract and in female αβERKO mice sex reversal was observed, it could be speculated that in the undifferentiated gonad lack of ER expression is required to induce testicular cell fate.

Based on the current knowledge, several issues remain to be elucidated, such as: (1) The effect of peripheral and/or locally produced estrogen on gonadotropin synthesis and/or secretion and immature germ-cell apoptosis and maturation during the life span; (2) Genomic or non-genomic pathways of estrogen receptor activation involved in the regulation of HPG axis and testicular physiology; (3) The specific role of ERα and ERβ in testicular physiology from infancy to adulthood; (4) Variability in the clinical phenotype and hormonal profile in men with ER and P450arom gene mutations, and (5) The role of ER activation pathways in the bipotential gonad cell fate differentiation in early embryogenesis.

Finally, this review provides a new insight related to estrogen synthesis and estrogen action in human physiology, especially in the male reproductive system at different periods of maturation, from infancy to young adulthood.

## Author Contributions

GG and NS contributed equally to the writing, review, and editing. MC, RM, and EB contributed to the writing and review. PR contributed to the figure design. AB conceived, wrote, reviewed, and determined the final version of the manuscript. All authors have contributed significantly, both related to their own scientific discipline and expertise, and to the overall manuscript and approved the final manuscript.

### Conflict of Interest

The authors declare that the research was conducted in the absence of any commercial or financial relationships that could be construed as a potential conflict of interest.
